# Cell non-autonomous requirement of p75 in the development of geniculate oral sensory neurons

**DOI:** 10.1038/s41598-020-78816-y

**Published:** 2020-12-17

**Authors:** Tao Tang, Christopher R. Donnelly, Amol A. Shah, Robert M. Bradley, Charlotte M. Mistretta, Brian A. Pierchala

**Affiliations:** 1 Department of Biologic and Materials Sciences, University of Michigan School of Dentistry, Ann Arbor, MI 48109 USA; 2 Department of Anatomy, Cell Biology & Physiology, Stark Neurosciences Research Institute, Indiana University School of Medicine, 320 West 15th Street, Indianapolis, IN 46202 USA; 3 Center for Translational Pain Medicine, Department of Anesthesiology, Duke University School of Medicine, Durham, NC 27710 USA

**Keywords:** Cell biology, Developmental biology, Neuroscience, Physiology, Medical research

## Abstract

During development of the peripheral taste system, oral sensory neurons of the geniculate ganglion project via the chorda tympani nerve to innervate taste buds in fungiform papillae. Germline deletion of the p75 neurotrophin receptor causes dramatic axon guidance and branching deficits, leading to a loss of geniculate neurons. To determine whether the developmental functions of p75 in geniculate neurons are cell autonomous, we deleted p75 specifically in Phox2b + oral sensory neurons (Phox2b-Cre; p75^fx/fx^) or in neural crest-derived cells (P0-Cre; p75^fx/fx^) and examined geniculate neuron development. In germline p75^−/−^ mice half of all geniculate neurons were lost. The proportion of Phox2b + neurons, as compared to Phox2b-pinna-projecting neurons, was not altered, indicating that both populations were affected similarly. Chorda tympani nerve recordings demonstrated that p75^−/−^ mice exhibit profound deficits in responses to taste and tactile stimuli. In contrast to p75^−/−^ mice, there was no loss of geniculate neurons in either Phox2b-Cre; p75^fx/fx^ or P0-Cre; p75^fx/fx^ mice. Electrophysiological analyses demonstrated that Phox2b-Cre; p75^fx/fx^ mice had normal taste and oral tactile responses. There was a modest but significant loss of fungiform taste buds in Phox2b-Cre; p75^fx/fx^ mice, although there was not a loss of chemosensory innervation of the remaining fungiform taste buds. Overall, these data suggest that the developmental functions of p75 are largely cell non-autonomous and require p75 expression in other cell types of the chorda tympani circuit.

## Introduction

During the development of the peripheral taste system oral sensory neurons of the geniculate ganglion project via the chorda tympani nerve to innervate taste buds and fungiform papillae in the anterior tongue, and via the greater petrosal nerve to innervate taste buds on the soft palate. Two neutrophins, brain-derived neurotrophic factor (BDNF) and neurotrophin 4 (NT-4), regulate the survival of geniculate ganglion neurons and promote their target innervation during development^1–9^. BDNF also maintains taste bud innervation and neuronal survival in a subset of oral sensory geniculate neurons in adulthood^6^.

Neurotrophins function via activation of two different classes of receptors, the Trk family of receptor tyrosine kinases and the p75 neurotrophin receptor (p75NTR). p75 is a single pass transmembrane receptor that belongs to the tumor necrosis factor (TNF) receptor superfamily. p75 interacts with TrkA, TrkB and TrkC that bind nerve growth factor (NGF), BDNF and NT-4, and neurotrophin 3 (NT-3), respectively^10^. p75 also interacts with Ret, the receptor tyrosine kinase for glial cell line-derived neurotrophic factor (GDNF) family ligands^11^. While p75 binds with high affinity to all four of the mature neurotrophins, it has significantly higher affinity for the proneurotrophins that act as pro-apoptotic and axonal pruning factors^12,13^. p75 signaling pathways play important roles in cell survival, apoptosis, neurite outgrowth and pruning, myelination, cell-cycle regulation and cell migration^14–17^. During embryonic development of the peripheral gustatory system, p75 initially regulates geniculate neuron axon guidance and branching, and subsequently promotes innervation of taste buds^18,19^. The failure of target innervation for most oral sensory geniculate ganglion neurons in *p75*^−*/*−^ mice leads to their programmed cell death, ultimately resulting in adult mice with a reduced complement of taste buds^20^. The physiologic consequences of this loss of geniculate neurons, taste buds and their innervation remain unknown.

p75 is highly expressed in migrating neural crest cells, glial cells and in most sensory neurons during development^14,15,21^. Germline deletion of p75 leads to the embryonic loss of 50% of DRG neurons across neuronal subtypes^22,23^. In contrast, deletion of p75 selectively from neurons in the DRG leads to a loss of 20% of adult DRG neurons, these losses belonging predominantly to the Ret + nonpeptidergic nociceptor population that emerges postnatally^11^. These findings suggest that much of the developmental effects of p75 relate to its function in non-neuronal cell types that contribute to the sensory circuit, such as Schwann cells or satellite glial cells. These observations in DRG neurons raise the question of whether the dramatic developmental deficits in geniculate neurons of the peripheral taste system are due to deficits in p75 signaling within geniculate ganglion neurons, or in other non-neuronal cell types that may contribute to target innervation and survival. To this end, we investigated whether the developmental functions of p75 in geniculate neurons are cell autonomous. We first evaluated *p75*^−*/*−^ mice and observed that approximately 50% of geniculate ganglion neurons were lost, both in the Phox2b + oral sensory population and in the Phox2b-pinna-projecting population. *p75*^−*/*−^ mice also exhibited profound deficits in chorda tympani responses to taste and tactile stimuli. However, p75 deletion from oral sensory neurons (Phox2b-Cre; p75^fx/fx^) did not alter the number of geniculate ganglion neurons, the innervation of taste buds, or the electrophysiologic responses to taste or lingual tactile stimuli. Collectively, these data suggest that like DRG sensory neurons, p75 functions in geniculate neuron development through a cell non-autonomous mechanism, requiring p75 expression in other cell types.

## Methods

### Mice

Cell type-specific *p75* mutants were produced by breeding mice with floxed *p75* alleles (*p75*^*fx/fx*^)^24^ with mice harboring the Cre-mediated recombination system driven by a Phox2b promoter (Phox2b-Cre; stock # 016223, Jackson Labs) or P0 promoter (P0-Cre; stock # 017927, Jackson Labs). The *p75*^*fx*^ allele was produced by flanking exons 4–6 with loxP sites, resulting in the deletion of the entire transmembrane and intracellular domains^24^ upon Cre recombination. The *p75*^−*/*−^ were produced in a similar manner in that exons 4–6 were deleted, eliminating any possibility of the expression of intracellular fragments^24^. All experiments were performed in compliance with the guidelines of the Association for Assessment and Accreditation of Laboratory Animal Care International (AAALAC) and approved by the Institutional Animal Care and Use Committees of the University of Michigan and Indiana University School of Medicine.

### Immunohistochemistry

Mice were euthanized, transcardially perfused with 4% paraformaldehyde (PFA), and isolated tissues were post-fixed in 4% PFA overnight at 4 °C. The tissue was transferred to 30% sucrose at 4 °C overnight for cryoprotection. Tissues were embedded in OCT (Tissue-TEK; VWR) and stored at − 80 °C until sectioned. Geniculate ganglions and tongues were sectioned at 20 or 25 μm on a cryostat (CM1950; Leica Biosystems) and mounted onto precleaned slides (Superfrost plus, ThermoFisher). Slides were washed in PBS for 15 min at room temperature and then blocked in 10% normal donkey serum (Jackson ImmunoResearch), 0.5% BSA (Sigma), and mouse-on-mouse blocking reagent (Vector Laboratories) in 0.3% Triton X-100/PBS (PBS-X). The sections were incubated with the following primary antibodies, which were diluted in 0.3% PBS-X with 0.5% BSA in a humidified chamber overnight at 4 °C: goat α-Phox2b (1:200; AF4940, R&D Systems), mouse α-Tuj1 (βIII-tubulin; 1:200; T8578 Sigma), rabbit α-P2X3 (1:200 to 1:400; AB5895, Millipore), rat α–cytokeratin-8 [TROMA-I; 1:100 (supernatant), DSHB], rabbit α-p75 (NGFr; 1:200; AB-N01AP, Advanced Targeting Systems), and chicken α-k5 (Keratin 5,1:100, BioLegend). The p75 antibody is targeted to amino acids 43–161 of the extracellular domain. After incubation in primary antibodies, the slides were washed four times in 0.3% PBS-X with 0.5% BSA, and were then incubated in secondary antibodies for 2 h at room temperature (1:200; donkey CF488, CF543, or CF633; Biotium). The slides were then washed three times and cover-slipped with DAPI Fluoromount-G (Southern Biotech).

### Imaging

Immunoreacted geniculate ganglia and taste buds were imaged using LAS-AF software on an SP5 confocal microscope (Leica Microsystems) or using LAS-X software on an SP8 Lightning confocal microscope (Leica Microsystems). Optical images were captured every 1 μm at either 20X or 63X magnification with high resolution (2048 × 2048). For all images, either two or three channels were imaged separately using single-wavelength excitation to generate a composite image.

### Quantification of fungiform papilla and taste bud innervation

Z-stacks were collected during confocal imaging such that fungiform papilla were captured in their entirely. During both image capture and analysis, the experimenter was naive to the mouse genotype. After the collection of images, they were imported into ImageJ using the Fiji image-processing package^25^. For the quantification of taste bud and fungiform papilla innervation, taste buds were outlined using K8 labeling to define the perimeter, and the basal epithelium defining the fungiform papilla core was used to outline the entire fungiform papilla. Once the border of taste bud and fungiform papilla regions were defined, the channels were split and converted to grayscale, and thresholds were set for each channel to minimize inclusion of nonspecific background. All of the Tuj1 + and P2X3 + pixels in K8 + and fungiform papilla regions were quantified by the software.

### Quantification of geniculate ganglion neurons

Intact geniculate ganglia were collected, cryo-sectioned (20 μm), imaged and processed as described above. The number of single labeled (Tuj1 +) and double labeled (Phox2b + and Tuj1 +) neurons were counted through each Z-stack section such that neurons were not counted more than once. All sections containing geniculate ganglion were counted and added together to determine the total number of neurons in the entire ganglion. The percentage of oral sensory neurons (Phox2b +) in the total population of geniculate neurons (Tuj1 +) was calculated and averaged for each mouse.

### Chorda tympani nerve recordings

Mice were anesthetized with an intraperitoneal (IP) injection of a ketamine/xylazine mixture (80–100 mg/kg ketamine, 5–10 mg/kg xylazine), and anesthesia was maintained with supplemental IP doses of ketamine (80–100 mg/kg). Recordings and stimulation sequences were as previously performed^26–28^. Briefly, the chorda tympani nerve was exposed through a lateral facial approach, cut centrally, and placed on a recording electrode, with a reference electrode placed on nearby tissue. Hypoglossal nerves were cut to prevent tongue movement. Amplified neural activity was monitored on an oscilloscope, was passed through to an analog-to-digital converter (Cambridge Electronic Design) and transferred to an integrator circuit. Tactile stimuli consisted of stroking the anterior tongue quadrant five times over a 5 s period. The chemical stimuli were placed onto the anterior tongue with a 5 ml syringe in the following order: 0.1, 0.5 M NaCl; 0.1, 0.5 M NH_4_Cl; 0.01 N HCl; 1.0 M sucrose; 0.04 M quinine HCl; 0.5 M NaCl; 0.1 M citric acid; 0.5 M MSG. Chemical solutions remained on the tongue for 10 s, followed by a distilled water rinse for at least 20 s. A concentration series of NaCl (0.05 M, 0.10 M, 0.25 M, 0.50 M, 1.00 M) was used to evaluate response–concentration effects. A series of chloride salts at 0.5 M (NaCl, KCl, NH_4_Cl, CaCl_2_, and MgCl_2_) were also applied. NaCl and NH_4_Cl stimuli (0.5 M) were applied three or four times during the entire nerve recording to monitor recording stability and baseline, and each series of chemical stimuli was repeated two or three times. For each individual chemical stimulus response, the height of the steady-state portion of the integrated response above the baseline was measured at 5 s after stimulus application. The absolute measurements of taste-evoked activity were used for statistical analyses and graphing for *p75*^−*/*−^ mice instead of a normalization to a standard stimulus, such as 0.5 M NH_4_Cl, because p75 knockout mice had essentially no responses to chemical stimuli.

### Statistical analyses

Results are expressed as mean ± standard error of the mean (SEM). The statistical tests were performed using two-way ANOVA with Tukey’s post hoc test to compare pairwise differences, or *t*-tests were performed, depending upon the number of groups, and we assumed equal variances to compare differences. The presence of asterisks indicates statistical significance: *, p < 0.05; **, p < 0.01; ***, p < 0.001. Sample sizes are indicated in the results and figure legends, and similar numbers of male and female mice were analyzed in all experiments.

## Results

### Oral sensory and pinna-projecting neurons are lost in geniculate ganglia of p75^−/−^ mice

**Figure 1:**
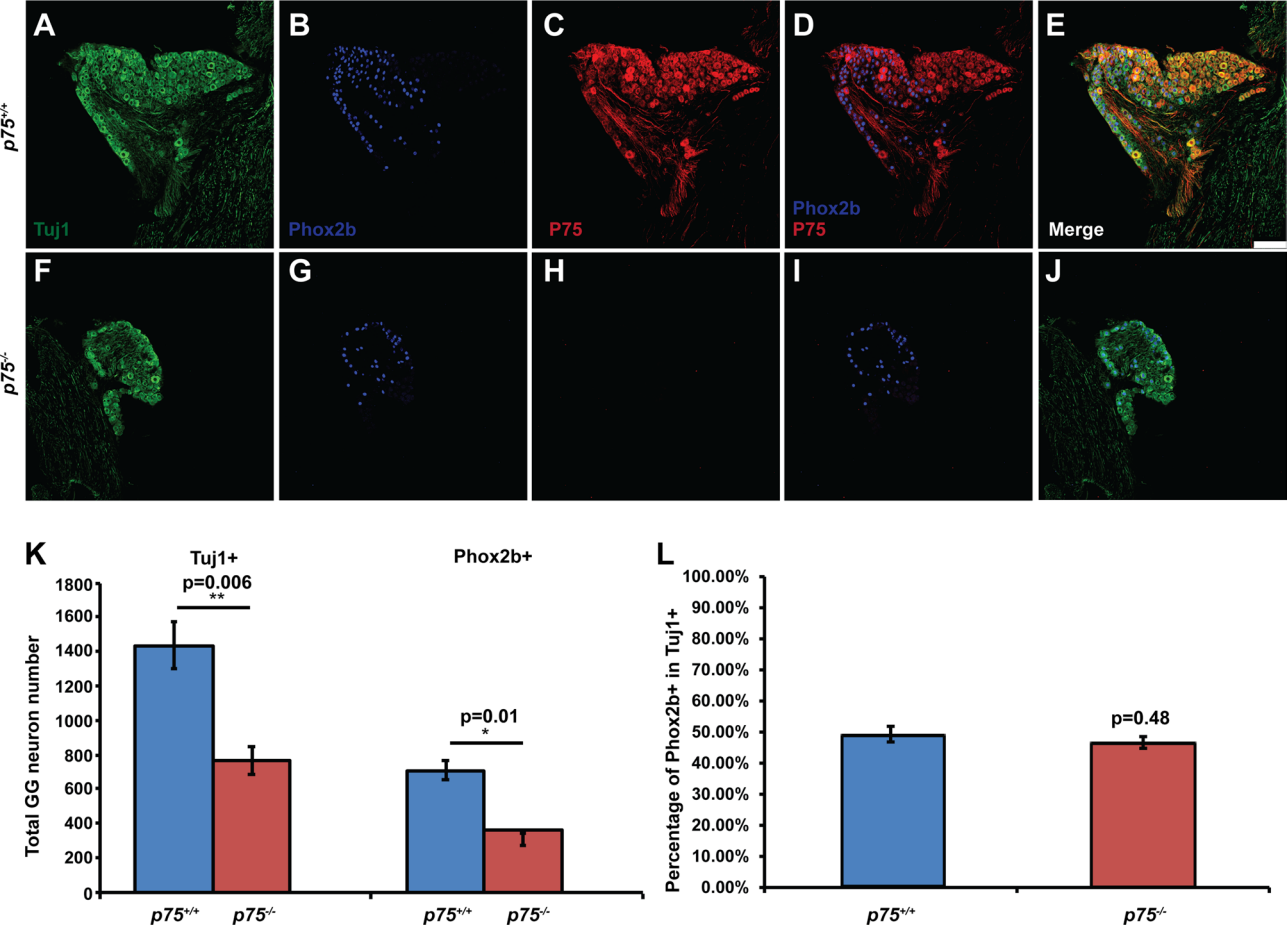
Both oral sensory and pinna-projecting somatosensory neurons are lost in the geniculate ganglia of *p75*^−*/*−^ mice. Geniculate ganglia from *p75*^+*/*+^ and *p75*^−*/*−^ mice were fixed and immunolabeled for Tuj1 (**A** and **F**, green, a marker of all neurons), Phox2b (**B** and **G**, blue, a marker of oral sensory neurons) and p75 (**C** and **H**, red). p75 was highly expressed in both Phox2b + and Tuj1 + /Phox2b-neurons in *p75*^+*/*+^ mice (**D**, **E**). No p75 immunolabel was observed in either Phox2b + or Tuj1 + neurons in *p75*^−*/*−^ mice (**I**, **J**), confirming p75 deletion. (**K**) The total number of Tuj1 + and Phox2B + neurons were quantified and displayed as a bar graph. The total numbers of geniculate ganglion neurons (Tuj1 +) were significantly reduced in *p75*^−*/*−^ mice (1434 ± 134) compared to *p75*^+*/*+^ mice (764 ± 54; p = 0.006), and the oral sensory (Phox2b +) neurons have a similar reduction in *p75*^−*/*−^ mice (708 ± 81) compared to *p75*^+*/*+^ mice (357 ± 32; p = 0.01). (**L**) The percentage of Phox2b + neurons was unchanged between *p75*^+*/*+^ and *p75*^−*/*−^ mice (p = 0.48), indicating both oral sensory and pinna-projecting somatosensory neurons (Phox2b-) are lost to a similar extent. The data are represented as the mean ± SEM; n = 3 for each group. Scale bar is 100 μm. * is p < 0.05, ** is p < 0.01.

A previous study examining the germline deletion of p75 in mice observed dramatic axon guidance and branching deficits of geniculate ganglion projections into the tongue at embryonic ages^18^. By adulthood 25–35% of geniculate ganglions neurons were lost in these p75^−/−^ mice^19,20^. The geniculate ganglion contains two populations of neurons, oral sensory neurons that project to taste buds and perigemmal cells in the anterior tongue and soft palate, and somatosensory neurons that innervate the pinna. To determine which population of neurons is most affected in *p75*^−*/*−^ mice, geniculate ganglion neurons expressing Tuj1 and Phox2b, defined as oral sensory neurons^29^, and pinna-projecting, somatosensory neurons that are Tuj1 + and lack Phox2b expression, were compared. We performed fluorescence immunolabeling for p75, Phox2b and Tuj1 in geniculate ganglia from *p75*^−*/*−^ and *p75*^+*/*+^ mice at 4–6 months of age. Note that for all experiments, the validity of Phox2b immunolabeling was confirmed by the presence of expression in autonomic ganglia, and the absence of staining in the trigeminal ganglion, as previously reported^29,30^. We observed p75 expression in the majority of both Phox2b + neurons and Tuj1 + /Phox2b-neurons in *p75*^+*/*+^ mice (Fig. 1A–E), while the p75 receptor was not observed in any geniculate neurons in *p75*^−*/*−^ mice (Fig. 1F–J). These findings confirmed that p75 was absent from *p75*^−*/*−^ mice, as reported previously^11,31^. The size of the geniculate ganglion was much smaller in *p75*^−*/*−^* mice*, as compared to *p75*^+*/*+^ mice, suggesting that neurons may be lost in *p75*^−*/*−^ mice. Indeed, we found that the total number of geniculate ganglion neurons (Tuj1 +) was significantly decreased in *p75*^−*/*−^ mice (764 ± 54) compared to *p75*^+*/*+^ mice (1434 ± 134; p = 0.006. Figure 1K). This finding is consistent with previous studies^19,20^. Oral sensory neurons (Phox2b +) had a similar reduction in *p75*^−*/*−^ mice (357 ± 32) compared to *p75*^+*/*+^ mice (708 ± 81; p = 0.012. Figure 1K). The proportion of oral sensory neurons (Phox2b +), as compared to the total number of geniculate ganglion neurons (Tuj1 +), was not different between in *p75*^−*/*−^ mice (46.66 ± 1.90%) and *p75*^+*/*+^ mice (49.33 ± 2.50%; p = 0.448. Figure 1L). Collectively, these data indicate that p75 deletion not only caused the loss of oral sensory neurons, but also pinna-projecting neurons of the geniculate ganglion.

### p75^−/−^ mice have impaired chorda tympani responses to taste and tactile stimuli

**Figure 2:**
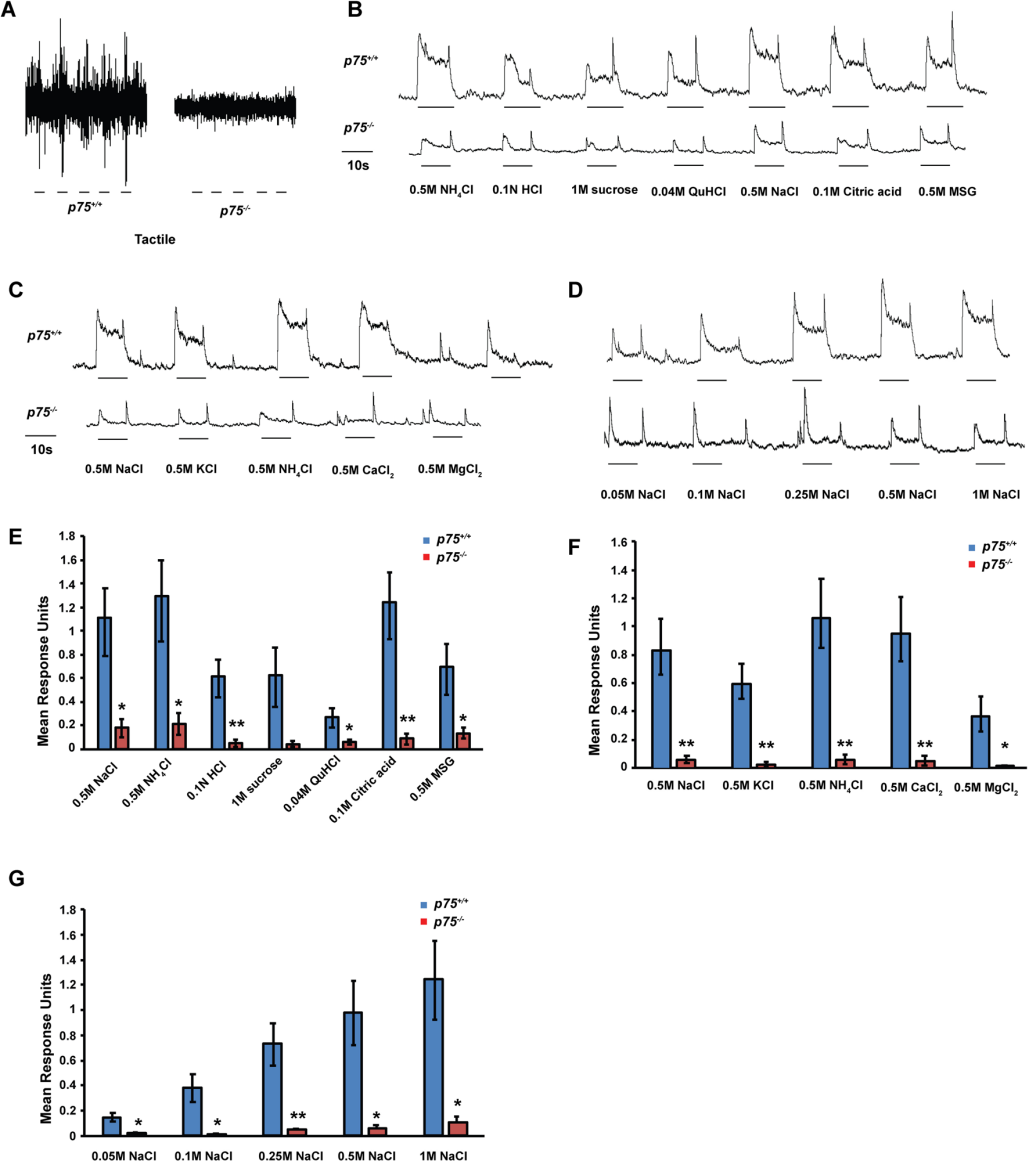
Both tactile and taste responses are reduced in *p75*^−*/*−^ mice. Whole nerve chorda tympani recordings were conducted on *p75*^+*/*+^ and *p75*^−*/*−^ mice at 6–8 months of age. Tactile stimuli, along with sweet, salty, bitter, sour and umami tastants, were applied to the tongues of these mice and the responses recorded. (**A**) These traces illustrate raw chorda tympani nerve responses to tactile stimuli. (**B**–**D**) Chemical taste responses are displayed as summated chorda tympani recordings to salt, acid, sweet, bitter and umami stimuli (**B**), several chloride salts (0.5 M, **C**) and a series concentration of sodium chloride (**D**). (**E**–**G**) Quantification of average absolute units of chorda tympani nerve responses (mean ± standard error) for *p75*^+*/*+^ and *p75*^−*/*−^ mice. (**E**) Salt (0.5 M NaCl), bitter (0.04 M QuHCl), sour (0.1 Citric acid) and umami (0.5 M MSG) responses were significantly reduced in *p75*^−*/*−^ mice. Sweet (1 M Sucrose) responses were not significantly different (p = 0.056) but showed a trend. (**F**) Chloride salt responses were all significantly reduced. (**G**) A concentration series of sodium chloride (0.05 M NaCl to 1 M NaCl) responses also displayed a significant reduction in *p75*^−*/*−^ mice. The traces (**A**–**D**) are representative responses from a mouse of each genotype, and n = 5 for both *p75*^+*/*+^ and *p75*^−*/*−^ mice (**E**–**G**). Scale bar is 10 s. * is p < 0.05, ** is p < 0.01.

To examine whether lingual sensory responses are also affected after p75 deletion, electrophysiological recordings from the chorda tympani were conducted. We applied tactile stimuli (stroking) and various chemical taste qualities (salts, acids, sucrose and quinine) to the tongues of *p75*^−*/*−^ and *p75*^+*/*+^ mice. There was a nearly complete loss of tactile responses from the chorda tympani in *p75*^−*/*−^ mice (Fig. 2A). Because tactile responses in the chorda tympani are rapidly adapting, the traces shown are raw data and are not summated. There was also a profound reduction in the chorda tympani responses to all tastants examined, and to an entire concentration range of sodium chloride, in *p75*^−*/*−^ mice as compared to *p75*^+*/*+^ mice (Fig. 2B–G). To quantify the magnitude of the reduction of nerve responses to tastants, we measured the height of the steady-state response above the baseline at 5 s after application of each stimulus. NaCl responses in *p75*^+*/*+^ mice increased as the applied concentrations increased, while *p75*^−*/*−^ mice exhibited a significant reduction in responses to all concentrations of NaCl (0.05 M NaCl, 0.02 ± 0.01 vs. 0.144 ± 0.034, p = 0.017; 0.1 M NaCl, 0.013 ± 0.003 vs. 0.378 ± 0.111, p = 0.023; 0.25 M NaCl, 0.047 ± 0.013 vs. 0.73 ± 0.168, p = 0.009; 0.5 M NaCl, 0.055 ± 0.027 vs. 0.976 ± 0.251, p = 0.014. 1 M NaCl, 0.11 ± 0.041 vs. 1.238 ± 0.314, p = 0.016. Figure 2H). Several chloride salts were applied to the tongues of *p75*^−*/*−^ mice, and they exhibited a significant reduction in response as compared to *p75*^+*/*+^ mice (0.5 M KCl, 0.025 ± 0.015 vs. 0.612 ± 0.123, p = 0.004; 0.5 M NH_4_Cl, 0.063 ± 0.033 vs. 1.092 ± 0.244, p = 0.007; 0.5 M CaCl_2_, 0.05 ± 0.033 vs. 1.082 ± 0.267, p = 0.009; 0.5 M MgCl_2_, 0.012 ± 0.002 vs. 0.38 ± 0.122, p = 0.033. Figure 2G). Average responses from *p75*^−*/*−^ mice also demonstrate significantly reduced responses for all taste qualities compared to *p75*^+*/*+^ mice (0.5 M NaCl, 0.178 ± 0.074 vs. 1.078 ± 0.286, p = 0.016; 0.5 M NH_4_Cl, 0.21 ± 0.091 vs. 1.256 ± 0.342, p = 0.018; 0.01 N HCl, 0.052 ± 0.024 vs. 0.6 ± 0.16, p = 0.009; 0.4 M QuHCl, 0.06 ± 0.023 vs. 0.266 ± 0.083, p = 0.037; 0.1 M Citric acid, 0.086 ± 0.043 vs. 1.208 ± 0.283, p = 0.005; 0.5 M MSG, 0.134 ± 0.045 vs. 0.676 ± 0.214, p = 0.033; Fig. 2F). The only exception was 1 M Sucrose, which showed a trend towards reduced responses in *p75*^−*/*−^ mice (p = 0.056). These data indicate that p75 is critical for geniculate ganglion neuron survival, as well as physiologic responses to mechanical and chemical sensory modalities in fungiform papillae and taste buds.

### Geniculate neurons are not lost in either Phox2b-Cre; *p75*^*fx/fx*^ or P0-Cre; *p75*^*fx/fx*^ mice

**Figure 3:**
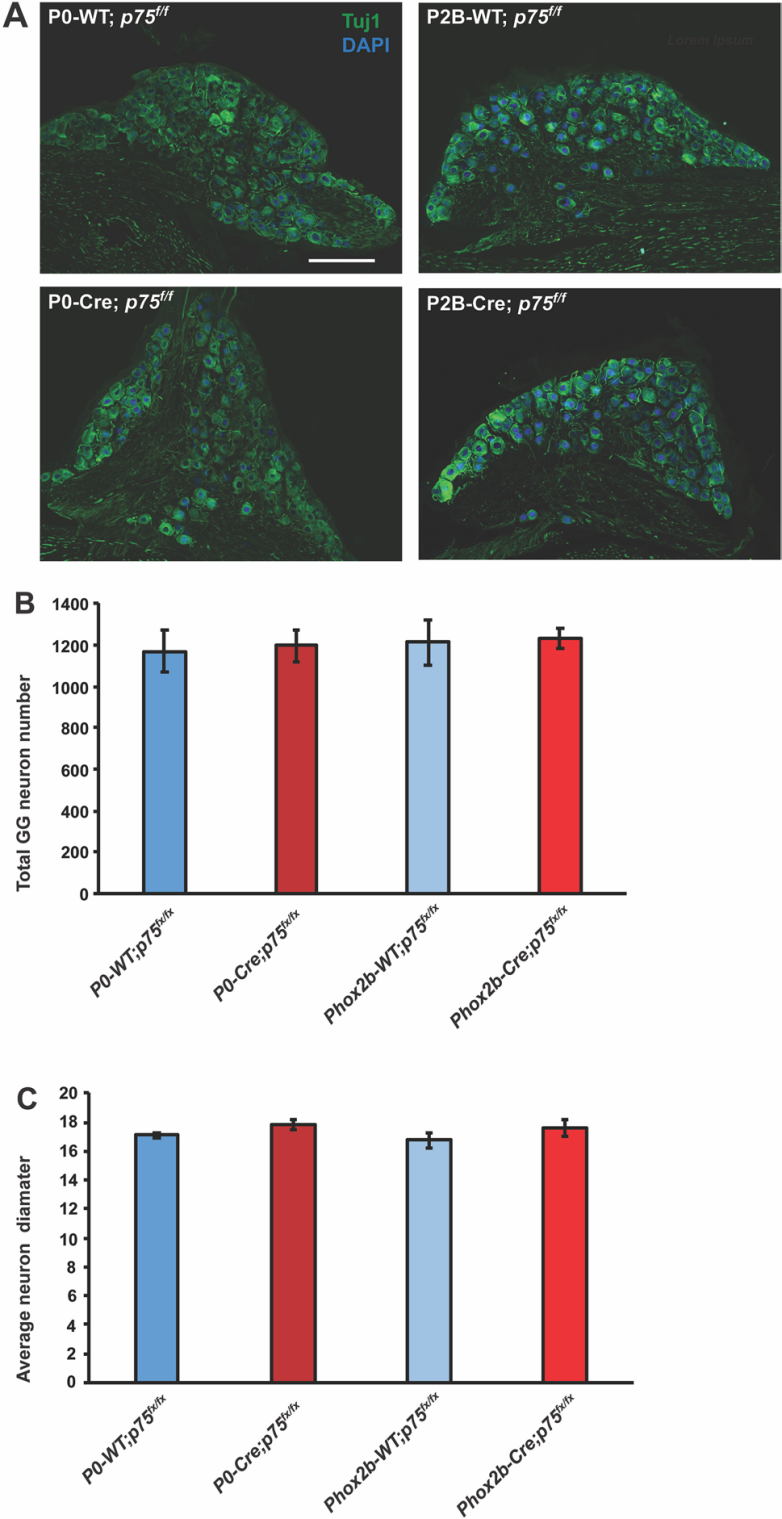
Geniculate ganglion neurons are not lost in either Phox2b-Cre; *p75*^*fx/fx*^ or P0-Cre; *p75*^*fx/fx*^ mice. (**A**) Geniculate ganglia from P0-Cre; *p75*^*fx/fx*^ mice, P0-WT; *p75*^*fx/fx*^ mice, Phox2b-Cre; *p75*^*fx/fx*^ mice and Phox2b-WT; *p75*^*fx/fx*^ mice were isolated and immunolabeled for Tuj1. (**B**) The total number of geniculate neurons was quantified from these images by counting Tuj1 + neurons when p75 was deleted specifically in Phox2b + oral sensory neurons (Phox2b-Cre; *p75*^*fx/fx*^) or in neural crest-derived cells (P0-Cre; *p75*^*fx/fx*^), and their wild type littermates, at 6–8 weeks of age. These data indicate that the total number of geniculate neurons does not change in either Phox2b-Cre; *p75*^*fx/fx*^ or P0-Cre; *p75*^*fx/fx*^ mice. (**C**) The somal diameters of geniculate neurons from Phox2b-Cre; *p75*^*fx/fx*^, Phox2b-WT; *p75*^*fx/fx*^, P0-Cre; *p75*^*fx/fx*^ and P0-WT; *p75*^*fx/fx*^ mice were measured. There were no differences in the average somal diameters among any of these groups. The data in this figure are represented as the mean ± SEM; n = 3–5 for each group.

A recent examination of p75 in the development of DRG sensory neurons revealed that neural deletion of p75 has a significantly milder effect on DRG survival than germline p75 deletion, suggesting that much of the developmental effects of p75 relate to its function in non-neuronal cell types^11^. To determine whether the developmental functions of p75 in geniculate neurons are cell autonomous, *p75*^*fx/fx*^ mice were crossed with Phox2b-Cre mice that express Cre in oral sensory neurons, or P0-Cre mice that express Cre in neural crest-derived cells. We first investigated how conditional deletion of the p75 receptor in Phox2b + neurons affected the number of geniculate ganglion neurons. We performed immunostaining to label Phox2b + and Tuj1 + geniculate ganglion neurons in Phox2b-Cre; *p75*^*fx/fx*^ and Phox2b-WT; *p75*^*fx/fx*^ mice, or in P0-Cre; *p75*^*fx/fx*^ and P0-WT; *p75*^*fx/fx*^ mice (Fig. 3A). The total number of Tuj1 + neurons was quantified from these mice at 8 weeks of age. We found no difference in the number of Tuj1 + neurons in either Phox2b-Cre; *p75*^*fx/fx*^ or P0-Cre; *p75*^*fx/fx*^ mice as compared to littermate control mice (Fig. 3B).

In addition to survival, neurotrophic factor receptor systems promote the growth and metabolic (trophic) status of neurons, which increases their somal size, among other morphological changes. To determine whether p75 deletion resulted in a decline in the somal size of geniculate neurons, the somal diameters were measured in Phox2b-Cre; *p75*^*fx/fx*^ mice, in P0-Cre; *p75*^*fx/fx*^ mice, and in their wild type littermates. There were no significant differences in the somal diameters of geniculate neurons from mice of any of these genotypes (Fig. 3C). Taken together, these results indicate that neuron-specific deletion, or neural crest-derived cell-specific deletion, of p75 did not impact geniculate ganglion neuronal survival or trophic status.

### Phox2b-Cre: p75^fx/fx^ mice have normal taste and tactile responses

**Figure 4:**
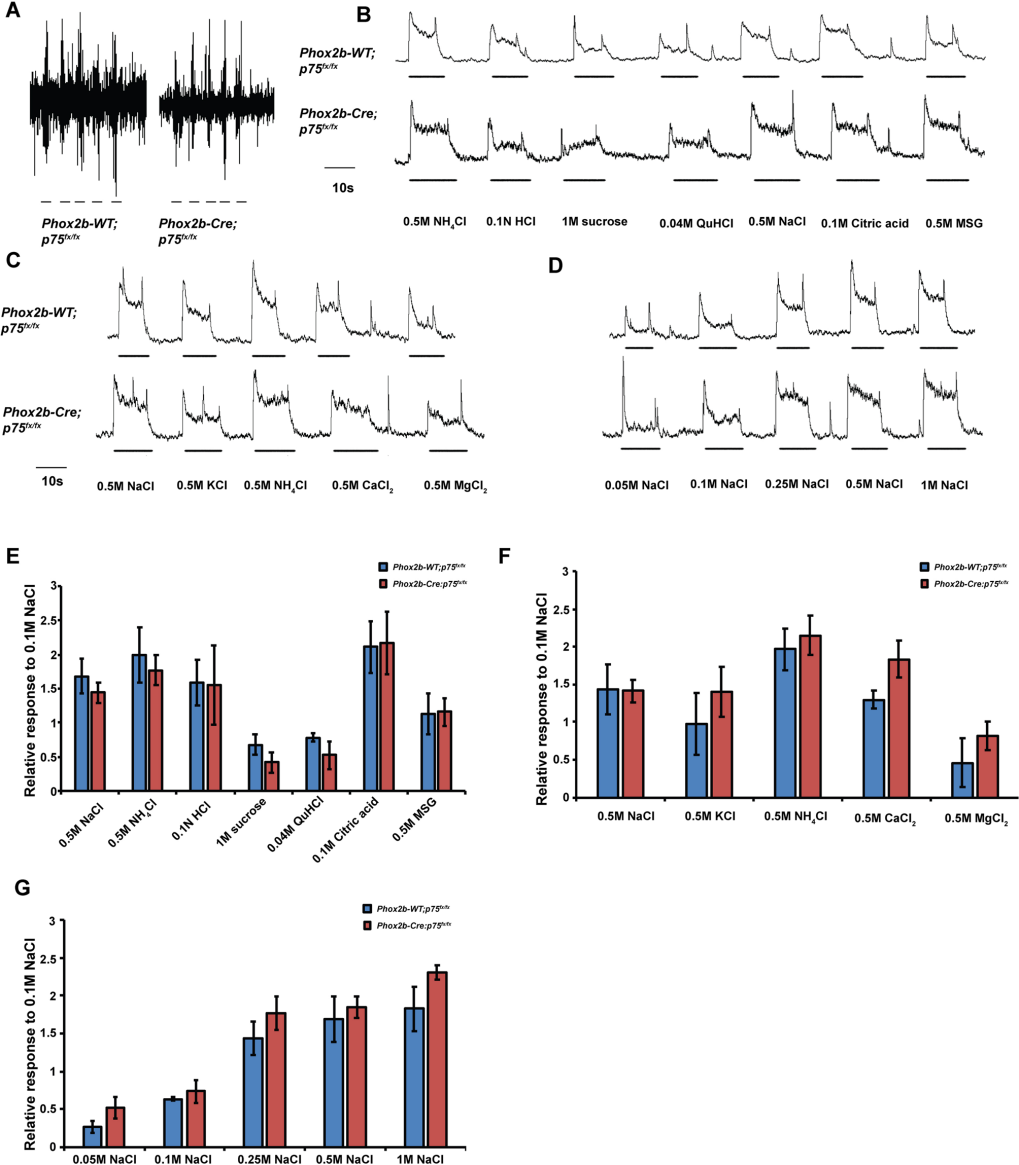
Deletion of p75 in oral sensory geniculate neurons does not affect taste or tactile chorda tympani responses. Representative raw (**A**) or summated (**B**–**D**) chorda tympani responses to tactile and taste stimuli, respectively, are displayed for Phox2b-WT; *p75*^*fx/fx*^ and Phox2b-Cre; *p75*^*fx/fx*^ mice. Chemical stimuli were quantified and graphed as relative responses compared to 0.1 M NaCl. There were no differences in responses between genotypes to NaCl, NH4Cl, HCl, sucrose, QuHCl, citric acid or MSG (**E**). There were also no differences in responses to chloride salts, or to a dose response curve to NaCl, between Phox2b-WT; *p75*^*fx/fx*^ and Phox2b-Cre; *p75*^*fx/fx*^ mice (**F**,**G**).

To determine whether p75 deletion in oral sensory neurons affected their electrophysiological responses to lingual stimuli, chorda tympani responses to both tactile and chemical stimuli were examined. Unlike p75^−/−^ mice, Phox2b-Cre; *p75*^*fx/fx*^ mice had tactile responses that were similar to Phox2b-WT; *p75*^*fx/f*x^ mice (Fig. 4A). Furthermore, responses to the 5 taste qualities examined were not statistically different between Phox2b-Cre; *p75*^*fx/fx*^ and Phox2b-WT; *p75*^*fx/fx*^ mice (0.5 M NaCl, 1.441 ± 0.146 vs. 1.685 ± 0.250, p = 0.463; 0.5 M NH_4_Cl, 1.772 ± 0.224 vs. 1.994 ± 0.412, p = 0.667; 0.01 N HCl, 1.553 ± 0.586 vs. 1.592 ± 0.34, p = 0.956; 1 M Sucrose 0.418 ± 0.15 vs. 0.675 ± 0.155, p = 0.285; 0.4 M QuHCl, 0.524 ± 0.206 vs. 0.782 ± 0.067, p = 0.230; 0.1 M Citric acid, 2.171 ± 0.467 vs. 2.116 ± 0.381, p = 0.930; 0.5 M MSG, 1.158 ± 0.204 vs. 1.123 ± 0.305, p = 0.654; Fig. 4B,E). There was also no difference in the chorda tympani responses to several chloride salts between these genotypes (0.5 M KCl, 1.395 ± 0.331 vs. 0.97 ± 0.408, p = 0.405; 0.5 M NH_4_Cl, 2.18 ± 0.26 vs. 1.963 ± 0.281, p = 0.721; 0.5 M CaCl_2_, 1.833 ± 0.246 vs. 1.297 ± 0.12, p = 0.224; 0.5 M MgCl_2_, 0.819 ± 0.187 vs. 0.457 ± 0.325, p = 0.166. Figure 4C,F). Because the electrophysiological responses to a concentration range of NaCl was similar in Phox2b-Cre; p75^fx/fx^ and Phox2b-WT; p75^fx/fx^ mice (Fig. 4D), we were able to calculate the responses to these chemical stimuli relative to the standard 0.1 M NaCl (Fig. 4E–G), unlike the analysis of *p75*^−*/*−^ mice in which all responses, including all concentrations of NaCl, were impaired. These data indicate that deletion of p75 in Phox2b + , oral sensory neurons does not impair their electrophysiological responses to tactile stimuli or tastants.

### The geniculate oral sensory neuron population is maintained in 12-month-old Phox2b-Cre: p75^fx/fx^ mice

**Figure 5:**
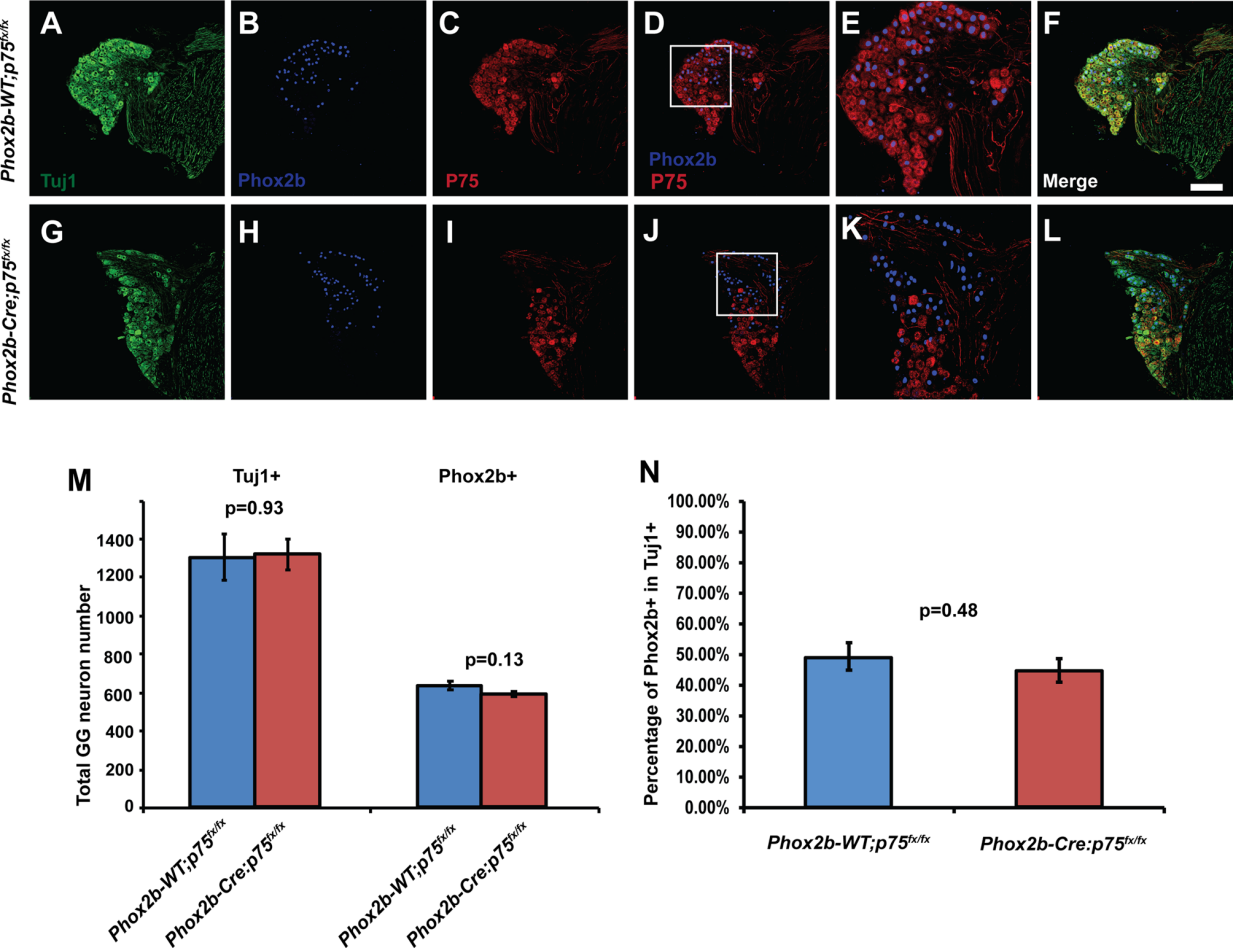
Geniculate neurons are not lost in 12-month-old Phox2b-Cre; *p75*^*fx/fx*^ mice. Geniculate ganglia from Phox2b-Cre; *p75*^*fx/fx*^ and Phox2b-WT; *p75*^*fx/fx*^ mice (9–12 months of age), were dissected and serially sectioned at 20 μm. Sections were immunolabelled for Tuj1 (green), p75 (red) and Phox2b (blue). In control (Phox2b-WT; *p75*^*fx/fx*^) mice we observed p75 labeling in both Tuj1 + and Phox2b + neurons (**C**–**E**). In the Phox2b-Cre; *p75*^*fx/fx*^ mice, p75 was not detected in Phox2b + neurons (**I**, **K**), but was observed in Tuj1 + /Phox2b-neurons (**J**, **K**), demonstrating that p75 was successfully deleted. Panels **E**, **F**, **K** and **L** display magnifications of the white boxes indicated in panels **D** and **J**. (**K**) The numbers of Tuj1 + and Phox2b + neurons were counted and graphed as the mean ± SEM. Quantifications indicated that total numbers of geniculate ganglion neurons (p = 0.93; n = 3) and the Phox2b + neurons (p = 0.13; n = 3) are unchanged. (**L**) Quantification of the percentage of Phox2b + neurons (compared to total Tuj1 + neurons) also exhibited no difference between groups (p = 0.48). Sample sizes are n = 3 for both genotypes; scale bar is 100 µM.

The observation that oral sensory neuron-specific and neural crest-specific deletion of p75 had no effect on geniculate neuron survival in young adult mice (8 weeks of age) was surprising, given the dramatic deficits in *p75*^−*/*−^ mice. After the period of programmed cell death in the geniculate ganglion, a population of neurons continues to require BDNF for phenotypic maintenance^9^. To determine whether p75 is required for maintenance of oral sensory neurons throughout the lifespan, geniculate ganglia from Phox2b-Cre; p75^fx/fx^ and Phox2b-WT; p75^fx/fx^ mice at 12 months of age were immunolabelled for Tuj1, p75 and Phox2b. In Phox2b-WT; *p75*^*fx/fx*^ mice we observed p75 immunolabeling that was co-expressed with both Tuj1 + /Phox2b + and Tuj1 + /Phox2b-neurons (Fig. 5A–F). In contrast, p75 immunolabeling was only observed in Tuj1 + /Phox2b-neurons, and was absent from Tuj1 + /Phox2b + neurons, in the Phox2b-Cre; *p75*^*fx/fx*^ mice (Fig. 5G–L). These data demonstrate that p75 was deleted selectively from oral sensory neurons (Phox2b +) in Phox2b-Cre; *p75*^*fx/fx*^ mice, and that geniculate neurons continue to express p75 in adulthood. During this analysis, no obvious difference was observed in the overall size of the geniculate ganglion across genotypes. To determine whether geniculate neuron numbers were altered upon p75 deletion, Tuj1 + neurons, Phox2b + neurons, and the proportion thereof, were quantified across genotypes. There were no significant differences in the number of Tuj1 + neurons (Phox2b-Cre; *p75*^*fx/fx*^ 1316 ± 81.265 vs. Phox2b-WT; *p75*^*fx/fx*^ 1302 ± 121.081; p = 0.929; Fig. 5M), Phox2b + neurons (Phox2b-Cre; *p75*^*fx/fx*^ 584 ± 12.17 vs. Phox2b-WT; *p75*^*fx/fx*^ 633 ± 32.605; p = 0.127; Fig. 5M), or in the proportion of Phox2b + neurons (Phox2b-Cre; *p75*^*fx/fx*^ 45 ± 3.82% vs. Phox2b-WT; *p75*^*fx/fx*^: 49 ± 4.46%; p = 0.48; Fig. 5N). There was also no difference in the somal diameters of 12-month-old Phox2b + neurons in the p75-deleted mice described above (data not shown). Collectively, these data indicate that postnatal expression of p75 is dispensable for the survival and trophic maintenance of oral sensory neurons up to 12 months of age.

The dramatic difference between the loss of geniculate neurons in *p75*^−*/*−^ mice as compared to Phox2b-Cre; *p75*^*fx/fx*^ mice raises the question of whether deletion of p75 in the Phox2b-Cre strain is sufficiently early in development. The p75-dependent loss of neurons in the geniculate ganglion begins at E14.5^18^, and Phox2b expression has been reported as early as E12.5^32,33^, suggesting deletion would occur during this period of cell death. In order to confirm that p75 deletion was robust during developmental ages, Phox2b-Cre; *p75*^*fx/fx*^, Phox2b-Cre; *p75*^*fx/*+^ and Phox2b-Cre; *p75*^+*/*+^ mice were analyzed between E14.5-P0. Geniculate ganglia were isolated and subjected to immunolabeling for p75, Tuj1 and Phox2b. In geniculate ganglia from Phox2b-Cre; *p75*^+*/*+^ mice (Supp. Figure 1A–E) and Phox2b-Cre; *p75*^*fx/*+^ mice (Supp. Figure 1F–J) p75 was expressed in essentially all Phox2b + neurons. In contrast, Phox2b + geniculate neurons from Phox2b-Cre; *p75*^*fx/fx*^ mice (Supp. Figure 1K–O) lacked p75 expression entirely. When a similar analysis was performed on P0 mice, Phox2b + geniculate neurons expressed p75 in Phox2b-Cre; *p75*^+*/*+^ mice (Supp. Figure 1A’–E’) and did not display p75 expression in Phox2b-Cre; *p75*^*fx/fx*^ mice (Supp. Figure 1F’–J’). These data indicate that p75 is efficiently deleted in developing mouse embryos by E14.5-P0, supporting the conclusion that p75 expressed in Phox2b + geniculate neurons is not required for its survival-promoting effects.

The mild phenotype of Phox2b-Cre; *p75*^*fx/fx*^ mice raises the question of where p75 is expressed in the lingual epithelium. In other parts of the periphery p75 is widely expressed in both neurons and non-neuronal cells developmentally. We examined the expression of p75 in the tongues of adult mice by immunolabeling for p75, K5 (a basal cell marker) and K8 (taste bud marker). We did not observe p75 + immunolabelling in taste bud cells (K8 region), or in the surrounding walls of the taste bud^19^, except in innervating taste bud fibers (Supp. Figure 2A–E). We also did not observe p75 expression in cells below the taste buds, where p75 expression has been reported in nestin-expressing stem cells^34^. It should be noted that there was extensive p75 expression in nerve fibers that were perigemmal and in the papilla-surround region, which made it difficult to discern between axonal labeling of fibers coursing over the various resident cells of the papilla, versus expression on the surface of the cells themselves (Supp. Fig. S2F–J). There were occasional K5 + cells that clearly co-expressed p75, but these cells were not observed in every papilla (Supp. Fig. S2F–J). Because p75 expression may change between developmental ages and adulthood, we examined p75 expression during the period of taste bud innervation and the differentiation of taste receptor subtypes at E18.5. Similar to the p75 immunolabeling in the adult, p75 was robustly expressed in axonal projections into and around taste buds, but not in the taste buds themselves (Supp. Figure 2A’–D’). There was frequent expression of p75 in cells in the papilla-surround regions, similar to that observed in the adult (Supp. Figure 2A’–D’). These observations suggest that, besides innervating axons, p75 is expressed in a subset of cells in the papilla-surround region, similar to the adult, but that they probably do not account for geniculate neuron innervation of taste buds given their sparseness and location.

### p75 expression in oral sensory neurons is required for the development of a subset of fungiform taste buds, but not for innervation of the remaining taste buds

**Figure 6:**
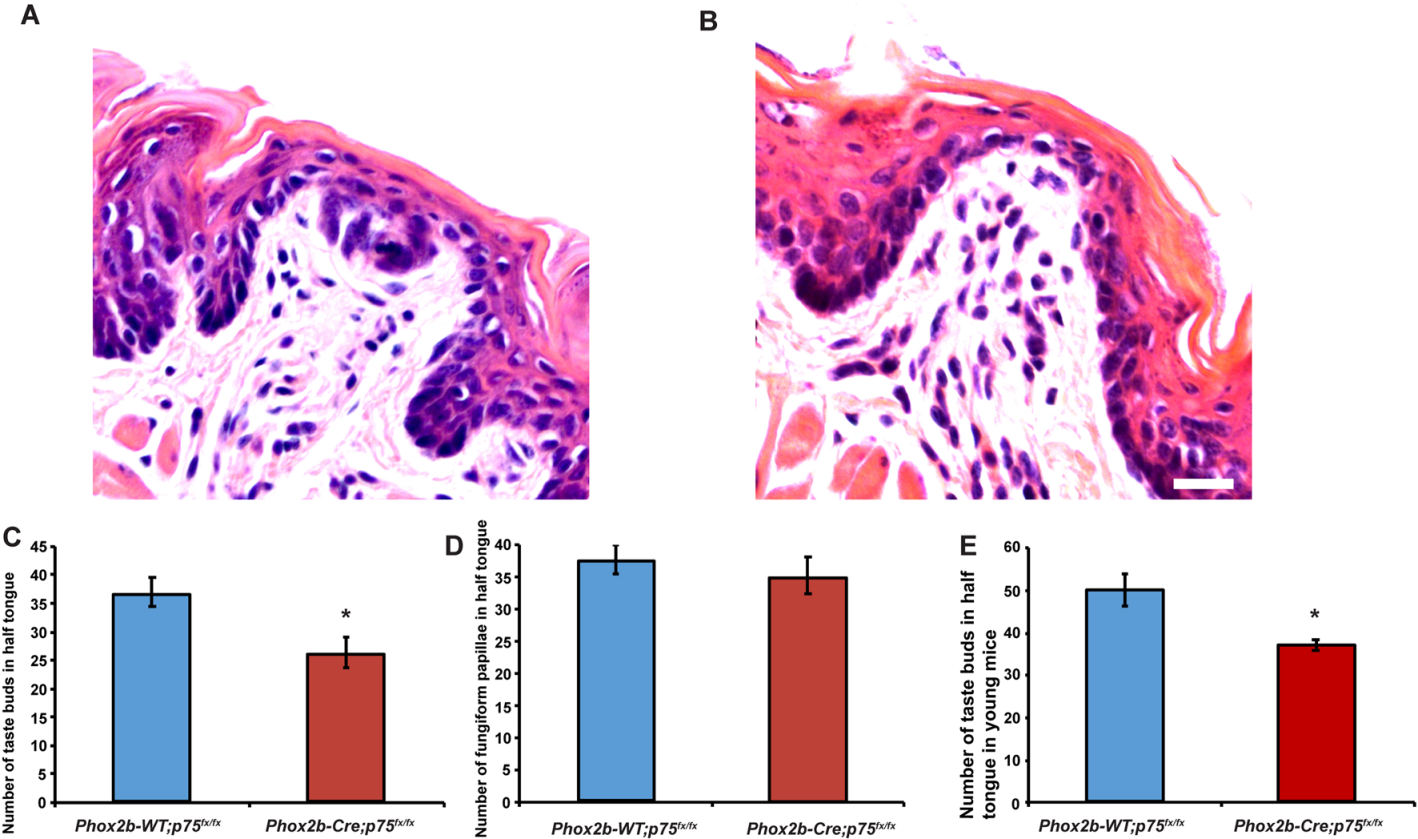
Neural deletion of p75 results in a moderate loss of fungiform taste buds. The tongues from 9–12-month-old Phox2b-Cre; *p75*^*fx/fx*^ and Phox2b-WT; *p75*^*fx/fx*^ mice were serially sectioned, H&E stained and the number of taste buds within fungiform papillae counted. While the majority of fungiform papillae contained taste buds with normal morphology (**A**), some had abnormal, shrunken taste buds or none at all (**B**). (**C**) Quantification of the number of taste buds revealed a reduction in Phox2b-Cre; *p75*^*fx/fx*^ mice compared to Phox2b-WT; *p75*^*fx/fx*^ mice. (**D**) Numbers of fungiform papillae were unchanged between genotypes. (**E**) The number of taste buds in 2–3-month-old Phox2b-Cre; *p75*^*fx/fx*^ mice was reduced as compared to Phox2b-WT; *p75*^*fx/fx*^ mice. Sample sizes are 3–6 mice per group and data are graphed as the mean ± SEM. * is p < 0.05. Scale bar in (B) is 25 μm.

Oral sensory neurons of the geniculate ganglion innervate taste buds in fungiform papillae located in the anterior region of the tongue. Although neural deletion of p75 did not alter electrophysiological responses to taste or tactile stimuli (Fig. 4), an extensive loss of taste buds must occur before deficits are observed in chorda tympani nerve recordings^35–37^. We therefore examined whether there were alterations in taste bud numbers and innervation in Phox2b-Cre; *p75*^*fx/fx*^ mice. For the quantification of the number of taste buds and fungiform papillae, H&E staining was performed on serial sections of tongues, and all of the fungiform papillae and taste buds were counted. While the majority of fungiform papillae contained morphologically normal taste buds, 15–20% of papillae contained an abnormal, shrunken taste bud or none at all (Fig. 6A and B). Comparing between Phox2b-Cre; *p75*^*fx/fx*^ mice with Phox2b-WT; *p75*^*fx/fx*^ mice, there were no significant differences in the total number of fungiform papillae (Phox2b-Cre; *p75*^*fx/fx*^ 35.25 ± 2.926 vs. Phox2b-WT; *p75*^*fx/fx*^ 37.75 ± 2.286; p = 0.525; Fig. 6D), but there was a significant loss of taste buds (Phox2b-Cre; *p75*^*fx/fx*^ 26.625 ± 2.688 vs. Phox2b-WT; *p75*^*fx/fx*^ 37 ± 2.549; 29% loss, p = 0.013; Fig. 6. C). To evaluate whether the loss of taste buds in Phox2b-Cre; *p75*^*fx/fx*^ mice occurs earlier in life, and is perhaps a developmental deficit, the number of taste buds were measured in young, 6 week Phox2b-Cre; *p75*^*fx/fx*^ and Phox2b-WT; *p75*^*fx/fx*^ mice. Similar to the older mice just described, there was a significant loss of taste buds in fungiform papillae, but not papillae themselves, from Phox2b-Cre; *p75*^*fx/fx*^ mice as compared to Phox2b-WT; *p75*^*fx/fx*^ mice (Fig. 6E).

**Figure 7:**
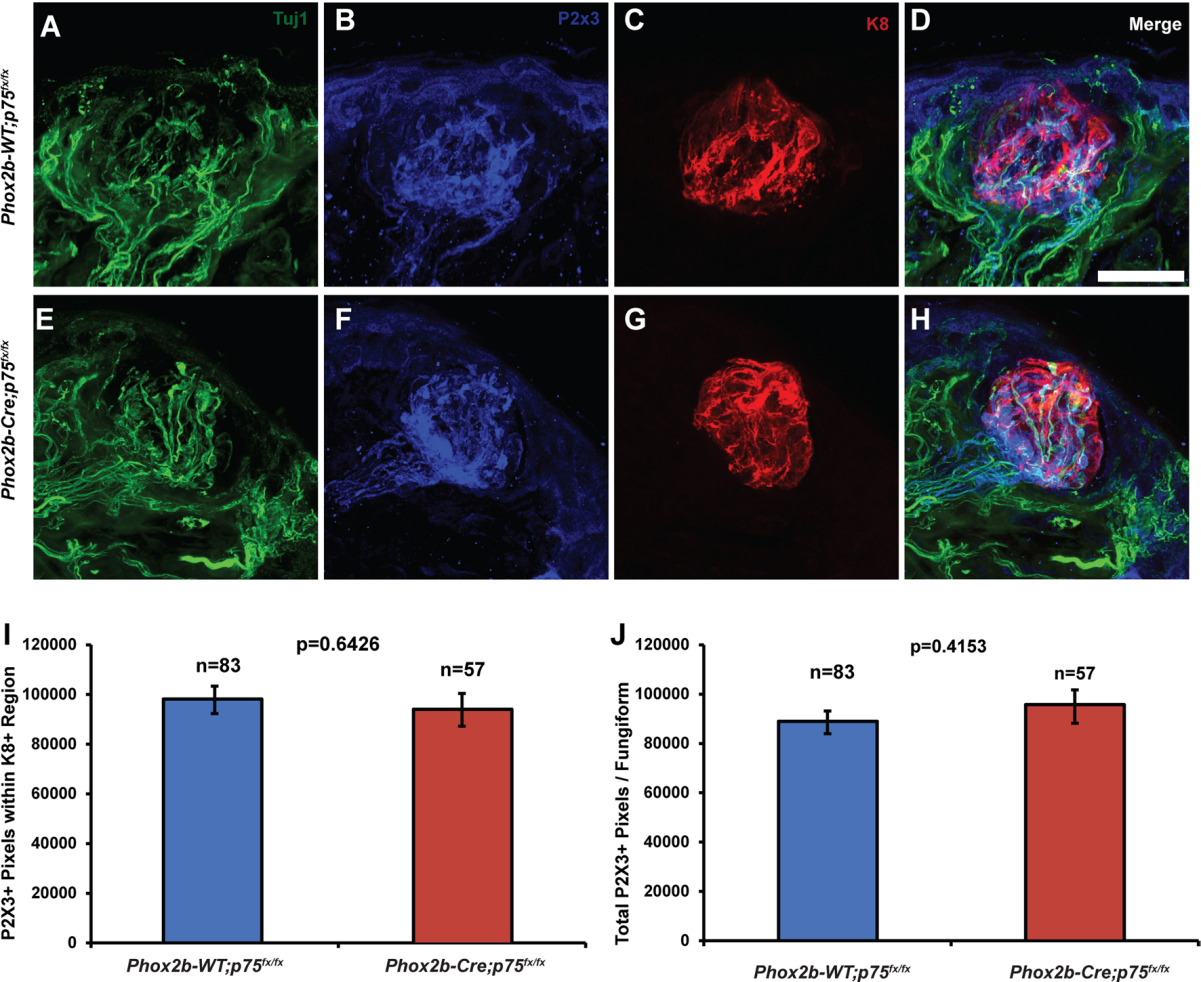
Innervation of fungiform taste buds is not altered in Phox2b-Cre; *p75*^*f/f*^ mice. The anterior tongues of Phox2b-Cre; *p75*^*fx/fx*^ and Phox2b-WT; *p75*^*fx/fx*^ mice were immunolabeled with antibodies to Tuj1 (green), P2X3 (blue) and K8 (red) to identify all axons, chemosensory axons and taste buds, respectively. (**A**–**D**) Tuj1, P2X3 and K8 immunolabeling of a typical fungiform taste bud from Phox2b-WT; *p75*^*fx/fx*^ mice. (**E**–**H**) The same immunolabeling method is shown in a typical fungiform taste bud from Phox2b-Cre; *p75*^*fx/fx*^ mice. Fungiform papillae were imaged from three independent mice per group (n = 83 taste buds in Phox2b-WT; *p75*^*fx/fx*^ mice and n = 57 taste buds in Phox2b-Cre; *p75*^*fx/fx*^ mice) and the amount of innervation measured. No differences were observed in either the number of P2X3 + pixels within K8 + regions (**I**; p = 0.64) or in the number of P2X3 + pixels within fungiform papillae (**J**, p = 0.42). Graphs indicate mean ± SEM. Scale bar = 25 μm.

To determine whether there was also a loss of taste bud innervation that could explain this deficit in the number of taste buds, tongues were immunolabelled with anti-P2X3 (marker of chemosensory axons and terminals), anti-Tuj1 (pan-axonal marker) and anti-K8 (marker for taste bud cells). Visual examination of P2X3 + and Tuj1 + fibers from Phox2b-Cre; *p75*^*fx/fx*^ and Phox2b-WT; *p75*^*fx/fx*^ mice did not reveal any obvious differences in the degree of taste bud innervation between these genotypes (Fig. 7A–H). The extent of P2X3 + and Tuj1 + innervation in taste bud (K8 labeled) regions was quantified in tongues from Phox2b-Cre; *p75*^*fx/fx*^ and Phox2b-WT; *p75*^*fx/fx*^ mice. Consistent with evaluation of the images, no differences were detected in the quantifications of P2X3-labeled and Tuj1-labeled nerve fibers within taste buds between genotypes (Fig. 7. I). When the amount of P2X3 + and Tuj1 + innervation was quantified within the fungiform papillae, there was also no statistically significant difference between Phox2b-Cre; *p75*^*fx/fx*^ and Phox2b-WT; *p75*^*fx/fx*^ mice (Fig. 7.J). Taken together, these data suggest that deletion of p75 specifically in Phox2b + neurons leads to an early and sustained loss of some fungiform taste buds, while not altering fungiform papilla numbers or the innervation of the remaining taste buds.

## Discussion

Peripheral gustatory neurons rely heavily on the neurotrophin BDNF and its receptor, TrkB, for survival, target innervation and phenotypic maintenance^2,3,5,6,8,38^. Because p75 has been described as a co-receptor along with Trks for the neurotrophins, it was not surprising that germline deletion of p75 results in a significant disruption in axon growth and branching, taste bud innervation and causes the loss of geniculate ganglion neurons^18–20^. Early studies of somatosensory DRG neurons in p75 knockout mice similarly demonstrated a 50% loss of neurons across subpopulations, including TrkA, TrkB and TrkC neurons^22,23^. A more recent evaluation of DRG neuron development in which p75 was deleted specifically in neurons (Islet1-Cre) found a considerably smaller neuronal loss. The Ret + nonpeptidergic nociceptor population was predominantly affected, and no loss of TrkA + or TrkB + neurons was observed^11^. Here we sought to determine whether p75 expression is required cell autonomously in oral sensory neurons of the geniculate ganglia for target innervation, survival and physiological responses to lingual stimuli. Analysis of p75 germline knock out mice confirmed that approximately 50% of geniculate neurons are lost equally in both oral sensory neurons (Phox2b +) and pinna-projecting somatosensory (Phox2b-) neurons. Consistent with these abnormalities, lingual chemical and tactile responses from the chorda tympani nerve were dramatically reduced in p75^−/−^ mice. In contrast, deletion of p75 from either oral sensory neurons (Phox2b-Cre) or neural crest-derived cells (P0-Cre) did not impact neuronal survival or electrophysiological responses to taste and tactile stimuli. While there was a reduction in the number of taste buds in fungiform papillae of Phox2b-Cre; *p75*^*fx/fx*^ mice, there was no detectable alteration in the innervation of the remaining taste buds. Consistent with these data, there were no observed deficits in chorda tympani nerve responses to taste and tactile stimuli. Taken together, these data suggest that p75 functions in a predominantly cell non-autonomous manner for the majority of its developmental functions in geniculate neurons.

The stark contrast between the phenotypes of *p75*^−*/*−^ mice compared to Phox2b-Cre; *p75*^*fx/fx*^ mice raises the question: in what cells is p75 acting to promote oral sensory neuron development? p75 has been reported to be expressed in taste buds and in cells making up the walls of fungiform papillae^19,34^. We did not observe significant p75 staining in taste buds, but there were occasional K5 + basal cells that expressed p75 within the papillae (Supp. Figure 2). P0-Cre; *p75*^*f/f*^ mice were examined, which deletes p75 from neural crest derivatives. While in the trunk neural crest cells give rise to peripheral neurons, Schwann cells, satellite glia in peripheral ganglia, and some sensory end organs, in the craniofacial region neural crest cells predominantly produce Schwann cells and brachial arch bone and cartilage. In the geniculate ganglion, both the neurons and satellite glia are derived from the neurogenic placodes. P0-Cre; *p75*^*f/f*^ mice did not display developmental deficits in geniculate chemosensory neurons, even though p75 is critical both for Schwann cell migration and myelination^39,40^. Taking cells of the geniculate ganglion itself into consideration, p75 expression was observed in the majority of cells and, after p75 deletion from Phox2b + cells, most pinna-projecting somatosensory neurons continued to express p75 (Figs. 1, 5). Because the geniculate ganglion is derived from the neurogenic placode, these cells would not be affected in the P0-Cre line, nor would satellite glial cells, which may participate in development of Phox2b + neurons in this structure. Overall, these data support the notion that the other cell types in which p75 functions are either in the geniculate ganglion itself or in peripheral cells other than Schwann cells.

During peripheral gustatory system development, p75^−/−^ mice displayed less geniculate ganglion axon branching, fewer taste buds and dramatically impaired taste bud innervation^18,20^. Upon examination of taste bud numbers and innervation in Phox2b-Cre: *p75*^*fx/fx*^ mice, we observed that 27% of taste buds were lost, but that innervation of the remaining taste buds and fungiform papillae was unchanged (Figs. 6, 7). This loss of taste buds may be due to early innervation deficits to a subset of taste buds that result in their degeneration by 6–8 weeks of age in Phox2b-Cre: *p75*^*fx/fx*^ mice (Fig. 6). Alternatively, p75 may be required in taste bud cells or fungiform papilla epithelial cells that are necessary for taste bud development, maintenance and renewal^41,42^. Interestingly, in Phox2b-Cre: *p75*^*fx/fx*^ mice, the loss of a subset of taste buds was not accompanied by disorganization of fungiform papillae or the appearance of cornified cells at the papilla apex (Fig. 6). This indicates that the impact of p75 deletion from oral sensory neurons was not as significant as lingual disruption of Hedgehog signaling in which the papillae undergo dramatic structural changes^35,36,43^. With Hedgehog signaling disruption, it is notable that whereas taste buds are lost, innervation remains^44^.

Previous studies of mice administered purinergic receptor inhibitors, or P2X2/P2X3 knockout mice, demonstrated a complete loss of chorda tympani responses to chemical tastants^45,46^. This elimination of ATP neurotransmission did not impair tactile or cold responses from the chorda tympani. Similarly, mice and rats subjected to inhibition of Hedgehog signaling with chemotherapeutic compounds such as LDE225 exhibited the profound loss of chemical, but not tactile, responses^33,36,37^. Coincident with the LDE225 treatment is the loss of taste buds, structural disorganization of fungiform papillae and a loss of chorda tympani nerve responses to tastants. Responses to tactile and cold responses remain, and chorda tympani innervation appears to remain near the prior location of the taste buds. Conversely, selective elimination of the Ret + subpopulation of geniculate neurons leads a dramatic reduction in tactile responses, but not chemical responses, in the chorda tympani^26^. Our analysis of *p75*^−*/*−^ mice demonstrated a profound reduction of both taste and tactile chorda tympani responses. These data suggest that the loss of taste bud innervation in *p75*^−*/*−^ mice affects all afferents coming from the chorda tympani, both taste and tactile responsive fibers. Therefore, while different classes of geniculate neurons may have selective mechanisms for the development and maintenance of their chorda tympani fibers, p75 function appears to be generally important for all chorda tympani projections. The identification of the cell types that p75 functions in for lingual geniculate innervation of taste buds is a future direction that will shed light onto the important contribution of non-cell autonomous mechanisms of oral sensory circuit development, and is likely to apply to other peripheral sensory systems.

## Supplementary information

Supplementary Information.

Supplementary Figure 1.

Supplementary Figure 2.

## References

[1] Krimm RF, Miller KK, Kitzman PH, Davis BM, Albers KM (2001). Epithelial overexpression of BDNF or NT4 disrupts targeting of taste neurons that innervate the anterior tongue. Dev. Biol..

[2] Patel AV, Huang T, Krimm RF (2010). Lingual and palatal gustatory afferents each depend on both BDNF and NT-4, but the dependence is greater for lingual than palatal afferents. J. Comp. Neurol..

[3] Nosrat IV, Agerman K, Marinescu A, Ernfors P, Nosrat CA (2004). Lingual deficits in neurotrophin double knockout mice. J. Neurocytol..

[4] Liebl DJ, Mbiene JP, Parada LF (1999). NT4/5 mutant mice have deficiency in gustatory papillae and taste bud formation. Dev. Biol..

[5] Nosrat CA, Blomlof J, ElShamy WM, Ernfors P, Olson L (1997). Lingual deficits in BDNF and NT3 mutant mice leading to gustatory and somatosensory disturbances, respectively. Development.

[6] Tang T, Rios-Pilier J, Krimm R (2017). Taste bud-derived BDNF maintains innervation of a subset of TrkB-expressing gustatory nerve fibers. Mol. Cell Neurosci..

[7] Runge EM, Hoshino N, Biehl MJ, Ton S, Rochlin MW (2012). Neurotrophin-4 is more potent than brain-derived neurotrophic factor in promoting, attracting and suppressing geniculate ganglion neurite outgrowth. Dev. Neurosci..

[8] Patel AV, Krimm RF (2012). Neurotrophin-4 regulates the survival of gustatory neurons earlier in development using a different mechanism than brain-derived neurotrophic factor. Dev. Biol..

[9] Huang T, Krimm RF (2014). BDNF and NT4 play interchangeable roles in gustatory development. Dev. Biol..

[10] Gentry JJ, Barker PA, Carter BD (2004). The p75 neurotrophin receptor: multiple interactors and numerous functions. Prog. Brain Res..

[11] Chen Z (2017). p75 is required for the establishment of postnatal sensory neuron diversity by potentiating ret signaling. Cell Rep..

[12] Lee R, Kermani P, Teng KK, Hempstead BL (2001). Regulation of cell survival by secreted proneurotrophins. Science.

[13] Kraemer BR, Yoon SO, Carter BD (2014). The biological functions and signaling mechanisms of the p75 neurotrophin receptor. Handb. Exp. Pharmacol..

[14] Chao MV (1994). The p75 neurotrophin receptor. J. Neurobiol..

[15] Hempstead BL (2002). The many faces of p75NTR. Curr. Opin. Neurobiol..

[16] Lopez-Sanchez N, Frade JM (2002). Control of the cell cycle by neurotrophins: lessons from the p75 neurotrophin receptor. Histol Histopathol.

[17] Meeker RB, Williams KS (2015). The p75 neurotrophin receptor: at the crossroad of neural repair and death. Neural Regen. Res..

[18] Fei D, Huang T, Krimm RF (2014). The neurotrophin receptor p75 regulates gustatory axon branching and promotes innervation of the tongue during development. Neural Dev..

[19] Fan L, Girnius S, Oakley B (2004). Support of trigeminal sensory neurons by nonneuronal p75 neurotrophin receptors. Brain Res. Dev. Brain Res..

[20] Krimm RF (2006). Mice lacking the p75 receptor fail to acquire a normal complement of taste buds and geniculate ganglion neurons by adulthood. Anat. Rec. A Discov. Mol. Cell Evol. Biol..

[21] Koike T (2019). Morphological characteristics of p75 neurotrophin receptor-positive cells define a new type of glial cell in the rat dorsal root ganglia. J. Comp. Neurol..

[22] Murray SS, Bartlett PF, Cheema SS (1999). Differential loss of spinal sensory but not motor neurons in the p75NTR knockout mouse. Neurosci. Lett..

[23] Lee KF (1992). Targeted mutation of the gene encoding the low affinity NGF receptor p75 leads to deficits in the peripheral sensory nervous system. Cell.

[24] Bogenmann E (2011). Generation of mice with a conditional allele for the p75(NTR) neurotrophin receptor gene. Genesis.

[25] Schindelin J (2012). Fiji: an open-source platform for biological-image analysis. Nat Methods.

[26] Donnelly CR, Shah AA, Mistretta CM, Bradley RM, Pierchala BA (2017). Biphasic functions for the GDNF-Ret signaling pathway in chemosensory neuron development and diversification. Proc. Natl. Acad. Sci. USA.

[27] Yokota Y, Bradley RM (2016). Receptive field size, chemical and thermal responses, and fiber conduction velocity of rat chorda tympani geniculate ganglion neurons. J. Neurophysiol..

[28] Yokota Y, Bradley RM (2017). Geniculate ganglion neurons are multimodal and variable in receptive field characteristics. Neuroscience.

[29] Ohman-Gault L, Huang T, Krimm R (2017). The transcription factor Phox2b distinguishes between oral and non-oral sensory neurons in the geniculate ganglion. J. Comp. Neurol..

[30] D'Autreaux F, Coppola E, Hirsch M-R, Birchmeier C, Brunet J-F (2011). Homeoprotein Phox2b commands a somatic-to-visceral switch in cranial sensory pathways. PNAS.

[31] Bogenmann E (2011). Generation of mice with a conditional allele for the p75(NTR) neurotrophin receptor gene. Genesis.

[32] Pattyn A, Morin X, Cremer H, Goridis C, Brunet JF (1997). Expression and interactions of the two closely related homeobox genes Phox2a and Phox2b during neurogenesis. Development.

[33] Dauger S (2003). Phox2b controls the development of peripheral chemoreceptors and afferent visceral pathways. Development.

[34] Mii S, Amoh Y, Katsuoka K, Hoffman RM (2014). Comparison of nestin-expressing multipotent stem cells in the tongue fungiform papilla and vibrissa hair follicle. J. Cell Biochem..

[35] Kumari A (2017). Recovery of taste organs and sensory function after severe loss from Hedgehog/Smoothened inhibition with cancer drug sonidegib. Proc. Natl. Acad. Sci. USA.

[36] Kumari A (2015). Hedgehog pathway blockade with the cancer drug LDE225 disrupts taste organs and taste sensation. J. Neurophysiol..

[37] Kumari A, Yokota Y, Li L, Bradley RM, Mistretta CM (2018). Species generalization and differences in Hedgehog pathway regulation of fungiform and circumvallate papilla taste function and somatosensation demonstrated with sonidegib. Sci. Rep..

[38] Patel AV, Krimm RF (2010). BDNF is required for the survival of differentiated geniculate ganglion neurons. Dev. Biol..

[39] Cosgaya JM, Chan JR, Shooter EM (2002). The neurotrophin receptor p75NTR as a positive modulator of myelination. Science.

[40] Bentley D, Lee KF (2000). p75 is important for axon growth and Schwann cell migration during development. J. Neurosci..

[41] Barlow LA (2015). Progress and renewal in gustation: new insights into taste bud development. Development.

[42] Mistretta CM, Kumari A (2017). Tongue and taste organ biology and function: Homeostasis maintained by Hedgehog signaling. Ann. Rev. Physiol..

[43] Ermilov AN (2016). Maintenance of taste organs is strictly dependent on epithelial Hedgehog/GLI signaling. PLOS Gen..

[44] Mistretta CM, Kumari A (2019). Hedgehog signaling regulates taste organs and oral sensation: distinctive roles in the epithelium, stroma, and innervation. Int. J. Mol. Sci..

[45] Finger TE (2005). ATP signaling is crucial for communication from taste buds to gustatory nerves. Science.

[46] Vandenbeuch A (2015). Postsynaptic P2X3-containing receptors in gustatory nerve fibres mediate responses to all taste qualities in mice. J. Physiol..

