# Supplementary material for: Cell non-autonomous requirement of p75 in the development of geniculate oral sensory neurons
Source: Sci Rep. 2020 Dec 17;10:22117. doi: 10.1038/s41598-020-78816-y (PMC7747618; doi:10.1038/s41598-020-78816-y)
Supplement: Supplementary file 1 — Supplementary Information. [file 41598_2020_78816_MOESM1_ESM.docx]

**Supplemental Information:**

**Cell non-autonomous requirement of p75 in the development of geniculate oral sensory neurons**

Tao Tang^1,2^*, Christopher R. Donnelly^1,3^*, Amol A. Shah^1^, Robert M. Bradley^1^, Charlotte M. Mistretta^1^ and Brian A. Pierchala^1,2#^

1. Department of Biologic and Materials Sciences, University of Michigan School of Dentistry, Ann Arbor MI, 48109

2. Department of Anatomy, Cell Biology & Physiology, Stark Neurosciences Research Institute, Indiana University School of Medicine, Indianapolis, IN 46202

3. Center for Translational Pain Medicine, Duke University School of Medicine, Durham, NC 27710

*These authors contributed equally

^#^Corresponding author:

Department of Anatomy, Cell Biology & Physiology

Indiana University School of Medicine

Stark Neurosciences Research Institute

320 West 15^th^ Street

Indianapolis, IN 46202

Email: [brpierch@iu.edu](mailto:brpierch@iu.edu)

**Supplemental Fig. 1: p75 deletion in Phox2b-Cre; *p75^fx/fx^* mice occurs during development.**

Geniculate ganglia were isolated from E14.5 (A-O) or P0 (A’-J’) mice, serially cryosectioned and immunolabeled for Tuj1 (green), Phox2b (red) or p75 (blue). Phox2b-Cre; *p75^+/+^* (A-E) and Phox2b-Cre; *p75^fx/+^* (F-J) ganglia displayed clear p75 labeling in Phox2b+ neurons, in contrast to Phox2b-Cre; *p75^fx/fx^* (K-O) ganglia that lacked p75 labeling in these neurons. These images (A-O) are from E14.5 embryos. An identical result was observed when geniculate ganglia from P0 Phox2b-WT; *p75^fx/fx^* (A’-E’) and Phox2b-Cre; *p75^fx/fx^* (F’-J’) mice were analyzed. These are representative images from n=3-4 littermate mice of each genotype.

**Supplemental Fig. 2: p75 expression in fungiform papillae.**

Tongues from adult wild type mice were immunolabeled with antibodies to K8 (red), K5 (green) and p75 (blue) and imaged at high magnification. (A-E) Immunolabeling revealed that the majority of p75 resided in axons that innervated both fungiform papillae surround regions and taste buds, suggesting that both chorda tympani and trigeminal nerve fibers express p75. (F-J) These images exemplify circumstances in which p75 labels nerve fibers coursing across cells in fungiform papillae (white arrowhead), and when p75 labeling is observed in non-neuronal cells near taste buds that were often K5+ (yellow arrowheads). (A’-D’) To examine p75 expression during development of taste buds, tongues from E18.5 embryos were immunolabeled with antibodies to K8 (red), Tuj1 (green) and p75 (blue). p75 predominantly labeled axon fibers (Tuj1+) and cells around the periphery of taste buds, similar to the localization observed in adult mice. These are representative images from n=3 mice, and tongues from *p75^-/-^* mice did not show p75 labeling.
