# Supplementary figures and images for: Cell non-autonomous requirement of p75 in the development of geniculate oral sensory neurons
Source: Sci Rep. 2020 Dec 17;10:22117. doi: 10.1038/s41598-020-78816-y (PMC7747618; doi:10.1038/s41598-020-78816-y)

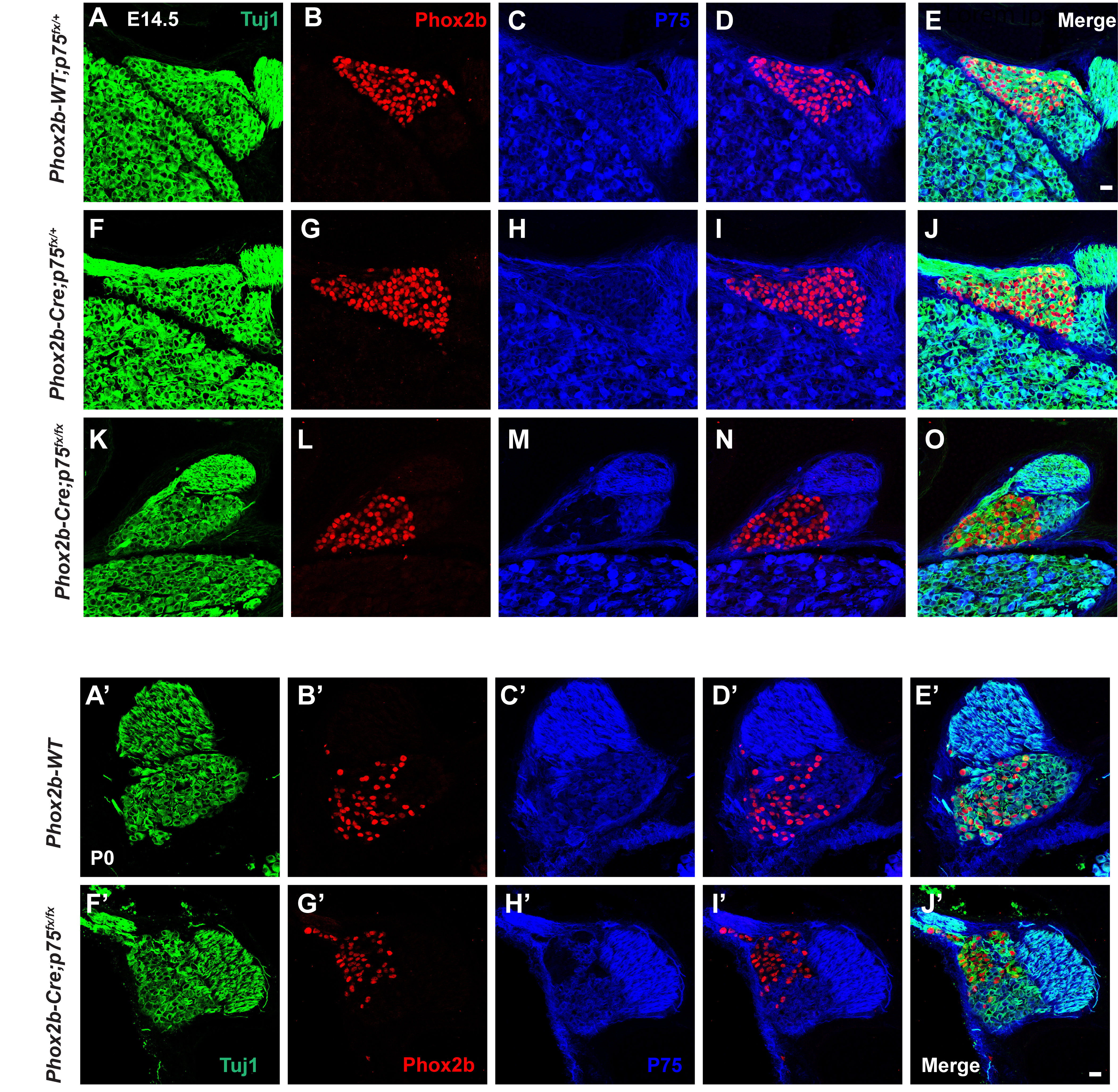

Supplement: Supplementary file 2 — Supplementary Figure 1. [file 41598_2020_78816_MOESM2_ESM.jpg]

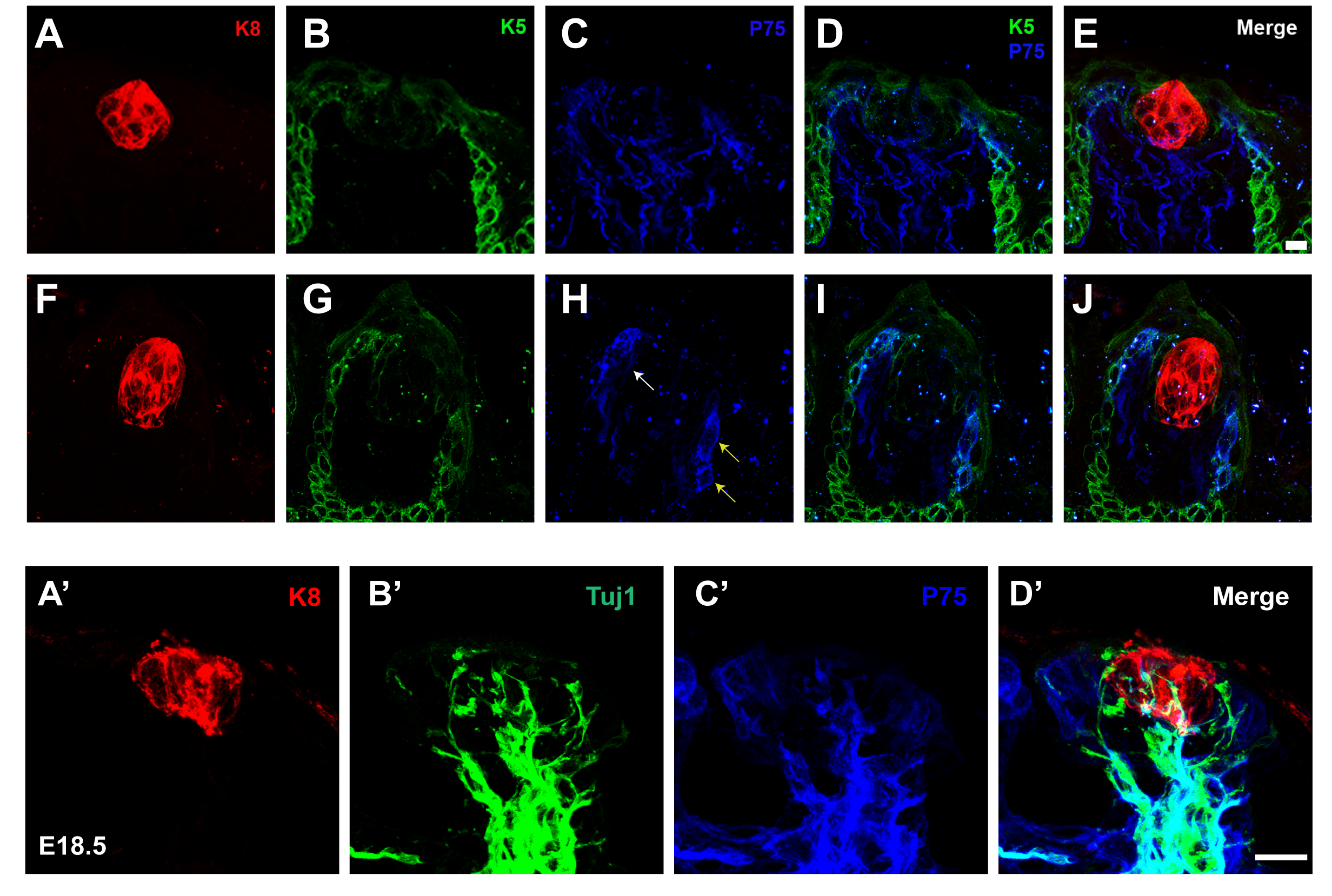

Supplement: Supplementary file 3 — Supplementary Figure 2. [file 41598_2020_78816_MOESM3_ESM.tiff]
